# Finite Element Simulation and Additive Manufacturing of Stiffness-Matched NiTi Fixation Hardware for Mandibular Reconstruction Surgery

**DOI:** 10.3390/bioengineering3040036

**Published:** 2016-12-19

**Authors:** Ahmadreza Jahadakbar, Narges Shayesteh Moghaddam, Amirhesam Amerinatanzi, David Dean, Haluk E. Karaca, Mohammad Elahinia

**Affiliations:** 1 Dynamic and Smart Systems Laboratory, The University of Toledo, Toledo, OH 43606, USA; ajahada@rockets.utoledo.edu (A.J.); Narges.ShayestehMoghaddam@rockets.utoledo.edu (N.S.M.); Amirhesam.Amerinatanzi@rockets.utoledo.edu (A.A.); 2 Department of Plastic Surgery, The Ohio State University, Columbus, OH 43212, USA; David.Dean@osumc.edu; 3 Department of Mechanical Engineering, The University of Kentucky, Lexington, KY 40506, USA; karacahaluk@uky.edu

**Keywords:** additive manufacturing (AM), superelastic NiTi, porosity, mandibular reconstructive surgery, finite element analysis, stiffness matching

## Abstract

Process parameters and post-processing heat treatment techniques have been developed to produce both shape memory and superelastic NiTi using Additive Manufacturing. By introducing engineered porosity, the stiffness of NiTi can be tuned to the level closely matching cortical bone. Using additively manufactured porous superelastic NiTi, we have proposed the use of patient-specific, stiffness-matched fixation hardware, for mandible skeletal reconstructive surgery. Currently, Ti-6Al-4V is the most commonly used material for skeletal fixation devices. Although this material offers more than sufficient strength for immobilization during the bone healing process, the high stiffness of Ti-6Al-4V implants can cause stress shielding. In this paper, we present a study of mandibular reconstruction that uses a dry cadaver mandible to validate our geometric and biomechanical design and fabrication (i.e., 3D printing) of NiTi skeletal fixation hardware. Based on the reference-dried mandible, we have developed a Finite Element model to evaluate the performance of the proposed fixation. Our results show a closer-to-normal stress distribution and an enhanced contact pressure at the bone graft interface than would be in the case with Ti-6Al-4V off-the-shelf fixation hardware. The porous fixation plates used in this study were fabricated by selective laser melting.

## 1. Introduction

Additive manufacturing (AM), also known as 3D printing, is useful for creating complex parts directly from Computer Aided Design (CAD) software. In general, all AM processes create physical parts directly from CAD data by adding material in successive layers. This layer-by-layer production process makes it possible to fabricate structures with engineered porosity. Current 3D printing devices used for the additive manufacturing of metals create layers by sintering or melting metal powders with a laser or an electron beam. These powder-bed technologies, such as Selective Laser Sintering (SLS), Selective Laser Melting (SLM), and direct metal laser sintering are the most common AM processes for AM fabrication of metal parts [1,2].

**Figure 1:**
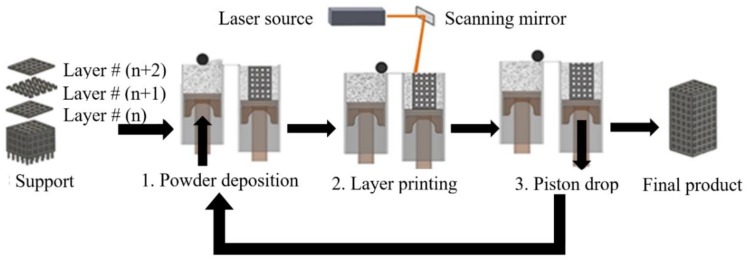
Additive Manufacturing (AM; 3D Printing) of metal parts.

Figure 1 illustrates the core components of conventional SLM or SLS 3D printing. First, the shape of the part as presented in the CAD file has to be converted into horizontal slices, layers that can be individually 3D printed. Subsequently, the file that contains the information for all of the layers is transferred to the AM machine. For powder-bed SLM processes, the powder material is spread by a blade, knife, or roller on a build plate. Then the laser melts and solidifies selective areas of the powder bed and creates the first layer of the part. After the fabrication of each layer, a new layer of powder is spread on top of the previous layer, and the laser sinters or melts the new layer onto the previously formed layer. The process is repeated until the fabrication process is completed. At the end of the process, the final part is surrounded by loose powder and the part is removed from the build plate [3].

NiTi is the most common shape memory alloy (SMA) showing unique functional properties, i.e., shape memory and superelasticity behavior [4,5]. Porous superelastic NiTi, in particular, has attracted much attention to be used in metallic implants due to their low stiffness close to that of cortical bone (i.e., 10–31.2 GPa), biocompatibility, hysteresis behaviors, and appropriate mechanical properties [1,6,7,8]. AM processes make it possible to fabricate implants with engineered porosity [9]. Recently, we have used SLM to create porous NiTi implants and medical devices [10,11,12,13,14,15,16].

Habijan et al. [17] have shown that the additively manufactured dense and porous NiTi samples are suitable carriers for human mesenchymal stem cells (hMSC) and the ion release (with the maximum concentration of 3.2 μg/L) is significantly below cytotoxic concentrations (25 mg/L). These achievements confirmed the biocompatibility and cytocompatibility of dense and porous NiTi [18]. These values are comparable to those obtained for the most common biocompatible material, i.e., Ti-6Al-4V [19].

It should be pointed out that the as-fabricated Ni-rich NiTi needs post-process heat treatments (i.e., solution annealing and aging) to acquire superelasticity behavior. In this study, we have completed a case study by using AM fabricated stiffness-matched porous NiTi skeletal fixation plates that would be useful in mandibular reconstructive surgery.

The standard of the care reconstructive surgery to repair a segmental defect of the mandible (lower jaw) involves the use of bone grafts, metallic fixation plates, and metallic screws to restore the mandible’s normal appearance and function (i.e., chewing, swallowing, breathing, and speech) [20]. Surgeons often use surgical grade five titanium, Ti-6Al-4V, fixation hardware in these procedures. That material has a much higher stiffness (112 GPa) than the surrounding cortical bone of mandible (10–31.2 GPa) [21,22]. Clinical reports show a high rate of complication from 7% to 69% after the surgery [23] and a low success rate, ranging from 34% to 64% [24,25]. In all cases, failed hardware necessitates removal surgery [26,27].

The high stiffness of Ti-6Al-4V fixation plates and screws can cause abnormal stress distribution throughout the mandible. Over time, this abnormal stress distribution may result in stress concentration in the fixation hardware and screws as well as stress shielding (i.e., reduced stress) of the cortical surface of the grafted bone and the host mandible. Stress-shielded bone may resorb, which may then lead to implant failure [28]. Literature reports resorption rates of 49.5% on the surrounding bone in the case of using traditional fixation plates within the first six months after grafting [29]. In addition to stress shielding, using a very stiff fixation device reduces the contact pressure between the graft/host bone, which may degrade the revascularization of the bone graft [30,31]. Traditionally, fixation plates are shaped (i.e., bent) in the operating room to fit the patient’s anatomy. This process increases the time and cost of the surgery as well as the risk to the patient [32,33]. However, 3D printed fixation plates can be bent to any degree determined in the implant CAD software prior to the surgery.

Using less stiff NiTi fixation plates may reduce stress shielding of grafted bone and elevate the compression force at the graft-host bone interfaces and subsequently enhance revascularization and healing of the grafted bone [34,35,36]. Furthermore, superelastic NiTi fixation plates provide the possibility of inducing tension on the mandible. To this end, the fixation plates undergo enough stress to enter the strain plateau before attaching to the host mandible. This solution is therefore expected to meet three requirements: first, to achieve a more natural stress distribution in the grafted and remaining host bone, thereby decreasing the risk of stress shielding; second, to increase the level of compressive pressure at the graft/host interfaces in order to enhance graft revascularization and defect site bone remodeling; finally, prefabricating a patient-specific fixation plate, which will fit directly on the patient’s mandible, reduces the time, cost, and risk of the surgery.

## 2. Finite Element Model for Implant Evaluation

The design of a patient-specific skeletal fixation device starts from Computed Tomography scan data (CT scan data) of the patient. CT scans use X-ray images taken from different angles to produce tomographic images [37]. The variation of local density of bone directly affects the resolution of the images in various zones. The high-resolution CT scan images of a patient’s mandible provide data about the geometric variables of the mandible and also the apparent density. The former data set provides the opportunity to create fixation plates based on a patient’s geometrical variation on the exterior surface of the mandible [38,39,40]. The latter data set allows estimation of the material properties of the surrounding bone using a quantitative scale method [41,42,43,44,45]. Knowledge of the exact geometry and the required fixation device stiffness allow the level of porosity to be determined in our CAD software.

**Figure 2:**
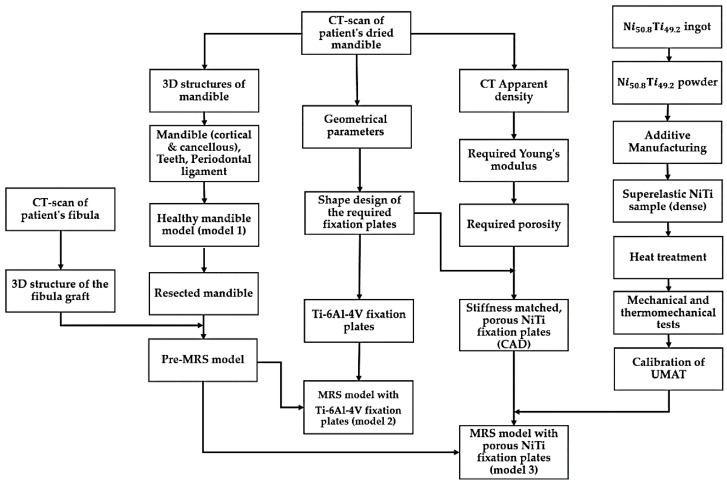
Flow chart for the design and fabrication of Ti-6Al-4V (standard of care) and NiTi stiffness-matched skeletal (i.e., mandible) fixation hardware. Finite Element Analysis models are used to evaluate the mandible and the fixation hardware during chewing (MRS = Mandibular Reconstructive Surgery, UMAT = User-defined Material subroutine).

This section highlights the development, calibration, and validation of a comprehensive model-based NiTi fixation hardware design work path as summarized in Figure 2. This general methodology can also be applied to design functional implants for other areas in the skeleton. Three different finite elements models were developed. A healthy mandible (model 1) and two Mandibular Reconstructive Surgery (MRS) simulated models, one with traditional dense Ti-6Al-4V (model 2) and another with stiffness-matched porous NiTi fixation plates (model 3). The first model simulates the highest bite force expected in a healthy patient with this particular patient’s anatomy. The part’s stiffness is then tuned to allow the desired stress distribution in the grafted bone and adjacent host mandible during maximum occlusal force. Sixty percent of the maximum bite force on the healthy mandible was incorporated into reconstructed models (i.e., model 2 and model 3 in Figure 2) [46]. The loads in both cases include muscle forces, bite forces, pre-tension and their combinations. The results are subsequently evaluated to show the performance of the fixation hardware in reducing stress shielding, stress concentrations, and improving contact pressure and reducing micro-motion at the host-graft bone interface. These are effective measures to reduce the risk associated with the mandibular segmental defect reconstructive surgery.

A CT scan (Aquilion 64; Toshiba Medical Systems, Tokyo, Japan) of a dry cadaveric human mandible was created to produce a 3D CAD file of all mandible components including cortical bone, cancellous bone, and the teeth. This device utilizes a 64-row Quantum detector, which enables the Aquilion CT scanner to acquire 64 simultaneous slices of 0.5 mm thickness with each 400 ms gantry revolution, resulting in precise isotropic imaging of the object. The gap between the teeth and the cortical bone was graphically filled with periodontal ligament tissue in ABAQUS (CAE v6.11, Dassault Systemes, Providence, RI, USA). For this study, a segment of the left half of the mandible bearing M1-3 (i.e., molar regions) was graphically resected with a length of 40 mm. To simulate a high fidelity reconstructive surgery, a double barrel fibular graft, inferior fixation plate, superior distal fixation plate, superior mesial fixation plate and screws were modeled and implanted in the resected region using SOLIDWORKS (Dassault Systèmes, Waltham, MA, USA).

**Figure 3:**
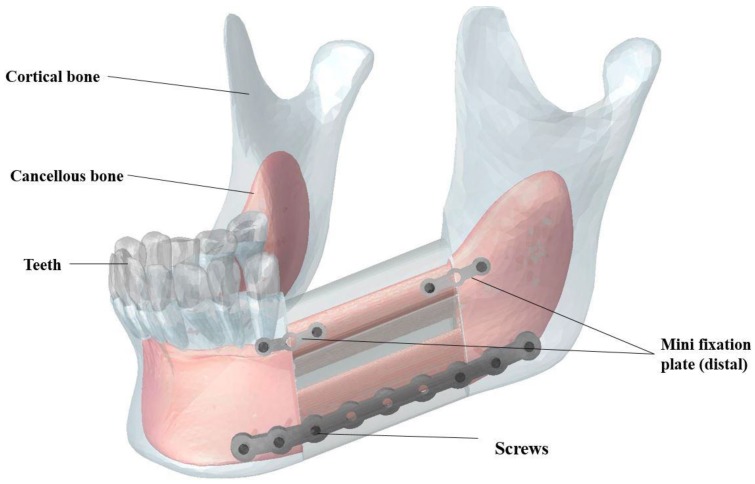
Components of the Reconstructed Mandible: double barrel fibular graft, one inferior fixation plate, one superior distal fixation plate, one superior mesial fixation plate, and fixation plate screws, cortical bone (**blue**) and cancellous bone (**red**).

As shown in Figure 3, the double barrel fibular graft was formed by virtually cutting the fibula of a patient. The two pieces were placed together to provide a total mesiodistal length of 40 mm, a buccolingual width of 14 mm, and a height of 38 mm. The inferior fixation plate and both superior fixation plates were equipped with nine and three threaded holes, respectively (see more details in Section 3). Six bicortical screws (i.e., long screws that pierce both the buccal and lingual cortical bone) and four unicortical screws (i.e., short screws that pierce the buccal cortical bone) were included in our Finite Element Analysis (FEA) to fix the inferior fixation plate and both superior plates, respectively [47,48]. The diameter of all screws was 1.4 mm.

All of the mandible graphical components were meshed in Hypermesh (Hyperworks, Troy, MI, USA) with 4-node tetrahedral elements (C3D4), which represent 3D, Solid, Tetrahedral, Deformable element type. The optimized number of elements is reported in Table 1 based on the mesh convergence analysis of each component.

All meshed parts were assembled, and constraints between screw-fixation plate, screw-host mandible, screw-fibular graft, teeth-ligaments and host mandible-ligaments were defined as tie constraints. It should be noted that the friction factors of 0 (simulation of the reconstructed mandible during the initial healing period) and 1 (simulation of the reconstructed mandible after the gaps between the grafted and host bone fragments had healed) are considered for simulation of surface to surface contact between host mandible and the fibular graft.

Table 2 summarizes the material properties of the FEA model components (i.e., fixation hardware, bone, teeth, and periodontal ligament) [1,49,50]. Cortical and cancellous bones were modeled as anisotropic material, and the other components were assumed to be linear elastic materials. It should be noted that Ti-6Al-4V material properties were assigned to the screws in all models.

The bite force created by masticatory muscles is the greatest source of stress that is usually applied to the mandible [4,51,52]. Our FEA models the peak masticatory load. Muscles are active even when the mandible is at rest. The amount of muscle forces depend on many factors including occlusion state (maximal chewing or soft food chewing) and bite loading conditions (balanced or unbalanced loading, bilateral or unilateral loading, grinding, or clenching) [53]. In this study, muscle forces related to a maximal bite force on the first right molar was considered as the loading input for the healthy mandible model (model 1) (i.e., a 526 N bite force based on Korioth et al. [46]). Three different fixation hardware scenarios (cases A, B, and C) were considered for the models 2 and 3.

**Figure 4:**
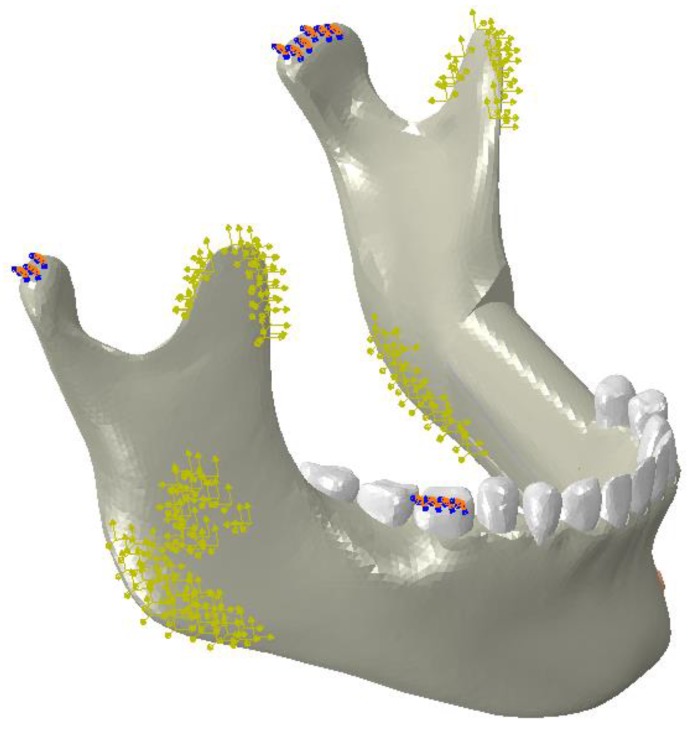
The finite element model of a mandible by considering the boundary conditions (**red** and **blue** points) and the muscle forces (**yellow** arrows).

Case (A): The simulation of the highest bite force after surgery (without applying any pretension to the fixation hardware). Since the reconstruction surgery causes a reduction in chewing power, all the muscle force values in this study were assumed to be of 60% of Korioth et al.’s [46] values for a normal healthy mandible [47]. This is the force applied in all three FEA models; Case (B): the simulation of used NiTi fixation hardware that had received 100 N of pretension load (i.e., on both superior and the inferior plates). This value for the pretension load was obtained from the FEA model in a way that increased contact pressure of the lower section of the graft bone by 50%; Case (C): this simulation used NiTi hardware that had undergone 100 N pretension load on both superior and the inferior fixation plates; More details on the model development and boundary conditions are presented in our previous studies [10,54,55] (Figure 4).

## 3. Fixation Hardware Design

CT-scan data were used in the design of the fixation plates (i.e., patient-specific fixation plates). This eliminates the need to bend fixation plates to fit the mandible during the surgery. In this study, the local lingual side curvature of each fixation plate was designed based on the patient’s mandible shape. The overall shape of the fixation plate was also designed to accommodate the insertion of screws at regular intervals and using commonly used thread geometries. The nine-hole inferior fixation plate has a buccolingual thickness of 1.5 mm, a mesiodistal length of 78 mm and a superoinferior width of 4 mm. Each of the two three-hole superior fixation plates has the dimensions of 1 mm × 18 mm × 2.8 mm, in buccolingual, mesiodistal and superoinferior direction, respectively. Six bicortical screws (i.e., long screws that pierce both the buccal and lingual cortical bone) and four unicortical screws (i.e., short screws that pierce the buccal cortical bone) were designed to fix the inferior fixation plate and both superior plates, respectively [47], to the host and graft bone. The diameter of all screws was 1.4 mm. The developed fixation plates and screws were assembled with the pre-MRS model component to simulate the MRS with traditional Ti-6Al-4V fixation plates (model 2).

**Figure 5:**
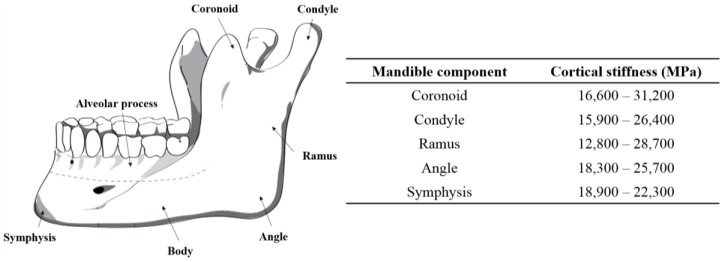
The stiffness in different regions of cortical bone the normal, adult mandible [1,46].

In addition to making sure that the fixation plate geometry ensures a good fit, directly on the surface of the host mandible and grafted fibular bones, this project also endeavors to ensure that the stiffness of this plate, and the screws holding it to the mandible and graft segments, have a stiffness that is matched with the stiffness of the cortical bone to which they are attached. The stiffness of cortical bone in the adult mandible varies between 10–31.2 GPa (Figure 5) [1]. The Young’s modulus for different zones of the mandible can be obtained using various methods, such as using a quantitative scaling technique to analyze CT-scan data of the patient’s mandible [41,42,43,44,45]. In our study, the estimated average Young’s modulus of the dried mandible in the cut segment (i.e., Molar regions) was calculated to be 12 GPa.

**Figure 6:**
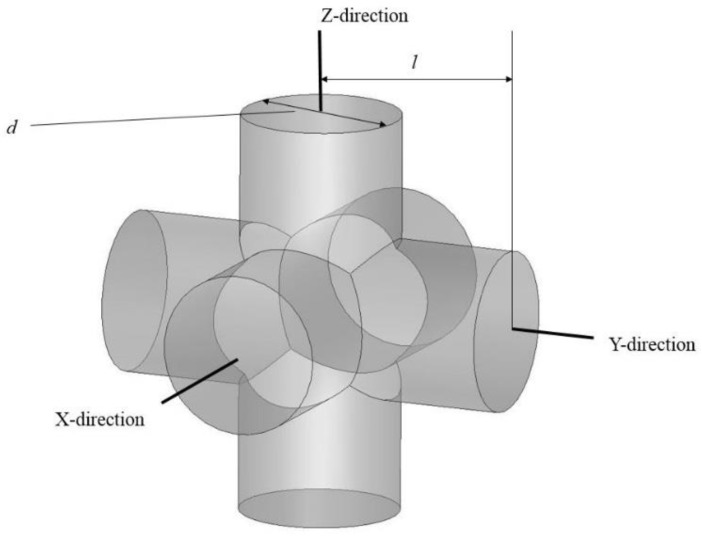
The unit pore cell that is used for our stiffness-matched NiTi fixation hardware. It is composed of three orthogonal hollow cylinders.

**Figure 7:**
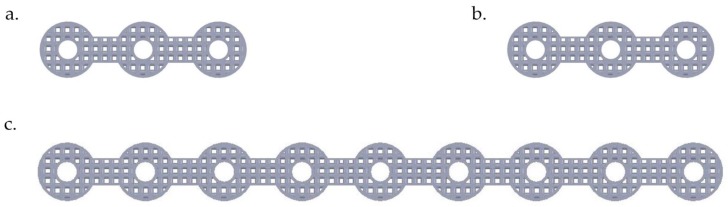
Porous fixation plates (i.e., superior mesial mini-plate (**a**), superior distal mini-plate (**b**), and inferior mandible bar (**c**)) with a porosity of 45.7%, created with an orthogonal cylinder geometry (see Figure 6). Note that all three plates would seat perfectly on the surface of the host and grafted bone that they will hold together.

The SLM-fabricated, dense NiTi samples still had a higher stiffness (37 GPa, measured in Section 4) than the estimated Young’s modulus of this mandibular cortical bone (i.e., 12 GPa). To this end, a porous NiTi part was created from a number of identical unit cells. Each unit cell was composed of three orthogonal cylinders, all with the same diameter, that intersect at their mid-point. Subsequently, a shell with the thickness of 0.2 mm was considered all around the fixation to smooth the edges. The level of porosity can be tuned by changing the diameter of the cylinders as shown in Figure 6. The level of porosity is calculated by dividing the volume of the porous unit sample to the volume of the surrounding cube. The equivalent stress on the porous part was calculated by dividing the axial force by the projected area of the unit cell on the plane normal to the loading direction. Additionally, the total displacement was used to calculate the equivalent strain. Based on the equivalent stress and strain of the porous structure, the equivalent Young’s modulus can be calculated. The equivalent stress is used to evaluate the performance of the device, and the actual stress was investigated to evaluate the safety of the fixation plate [11] (i.e., will it be strong enough during the healing period). It was thus calculated that 45.7% porosity is required to achieve an equivalent Young’s modulus of 12 GPa in the first fixation plate that we designed. A useful diameter in the fixation plate pore unit cells was calculated to be one millimeter. This porous structure, as Figure 7 shows, was created through imposing unit pore cells throughout the designed fixation plate. This porous hardware was assembled with the pre-MRS model component (model 3) for comparative studies.

## 4. Fabrication of Patient-Specific NiTi Fixation Hardware

In order to calibrate our FEA model to simulate NiTi fixation plates and screws, we fabricated dense compression specimens, performed heat treatments, and performed mechanical and Differential Scanning Calorimetry (DSC) tests to obtain the required material properties [56,57,58,59]. To this end, ${\mathrm{Ni}}_{50.8}{\mathrm{Ti}}_{49.2}$ (at %) ingots from NiTi Devices & Components, Inc. (Fremont, CA, USA), were atomized to powder using an Electrode Induction-melting Gas Atomization (EIGA) technique (by TLS Technique GmbH (Bitterfeld, Germany)). A PXM 3D printer (3D Systems, Rock Hill, SC, USA) equipped with a 300 W Ytterbium fiber laser for SLM was used in this study to fabricate a fully dense cylindrical shapes of 8 mm in diameter and 12 mm in length. The laser had a beam quality of $M^{2}<1.2$ and a Gaussian beam profile (TEM00). The optimized parameters of fabrication of these Ni-rich NiTi parts were from the same as in studies previously published by our group [2,13]. Those process parameters are summarized in Table 3. Finally, the designed porous superelastic NiTi fixation plates were fabricated based on the optimal parameters [10,60,61,62,63,64,65].

After the fabrication process, the cylindrical samples were solution annealed (1223 K, 5.5 h, H_2_O), aged (623 K, 15 min), and then water quenched. To perform solution annealing, Lindberg/Blue M BF514541 Box furnace was used. To avoid oxidation of the samples, they were placed in an argon-filled quartz ampules. For determining the transformation temperatures (TTRs), a Perkin-Elmer Pyris 1 DSC with the heating/cooling rate of 10 °C/min in a nitrogen atmosphere was used. The samples were loaded up to 800 MPa (before reaching to the critical stress for plastic deformation) and unloaded using a 100 kN MTS Landmark servo-hydraulic test platform (Minneapolis, MN, USA). The strain rate of 10^−4^·s^−1^ was employed during loading, whereas unloading was performed under force control at a rate of 100 N·s^−1^. An MTS high-temperature extensometer was used to obtain strain measurements [56]. The observed mechanical properties of these SLM fabricated samples are shown in Table 4. The impurity limits for medical NiTi are prescribed in ASTM F2063-05. The impurity level for clinical applications must be below 0.05 ppm [62]. The impurity content was tested in three of the NiTi samples that we fabricated. We observed impurity levels of 0.035, 0.0500, and 0.007 ppm for carbon, oxygen, and nitrogen, respectively, for the sample with the highest level of impurity.

An ABAQUS user-defined material (UMAT) subroutine based on the microplane theory [67,68,69] was calibrated using the experimental data of the fabricated NiTi parts. The UMAT is capable of simulation of both the superelastic and the shape memory effects of NiTi under multiaxial loading conditions regardless of the geometry of structure [70]. In order to calibrate the UMAT, the required parameters were extracted from mechanical (i.e., compression test at different temperatures) and thermomechanical experiments (i.e., DSC tests). More details on our UMAT calibration testing procedures are presented elsewhere [71].

FEA was used to determine the stiffness needed to stiffness-match the 3D printed fixation hardware. The final design of the porous NiTi fixation plates consisted of a desired level percentage of porosity, pore shape and size, and general dimension of the plate. The resulting CAD file of the designed NiTi fixation plate was used on the SLM machine for fabrication. To induce superelasticity, the same heat treatment procedures including solution annealing (1223 K, 5.5 h, H_2_O) and aging (623 K, 15 min) were performed.

## 5. Validation and Results

**Figure 8:**
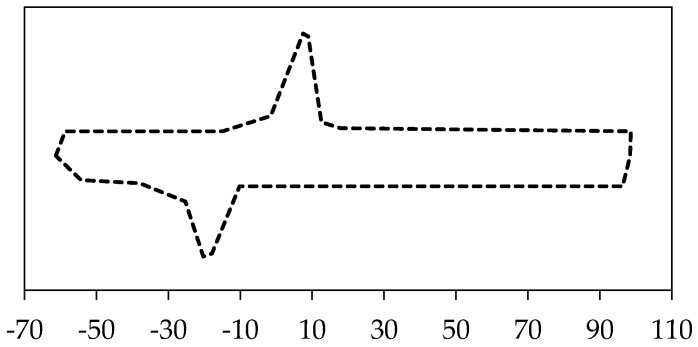
Differential Scanning Calorimetry (DSC) test for the 100% dense NiTi samples.

The input parameters of the UMAT were found from the DSC and thermomechanical tests on dense cylindrical samples in order to calibrate, tune, and finally, validate the model. The required parameters for calibration are summarized in Table 5 [71]. Transformation temperature has been measured using DSC test (Figure 8).

**Figure 9:**
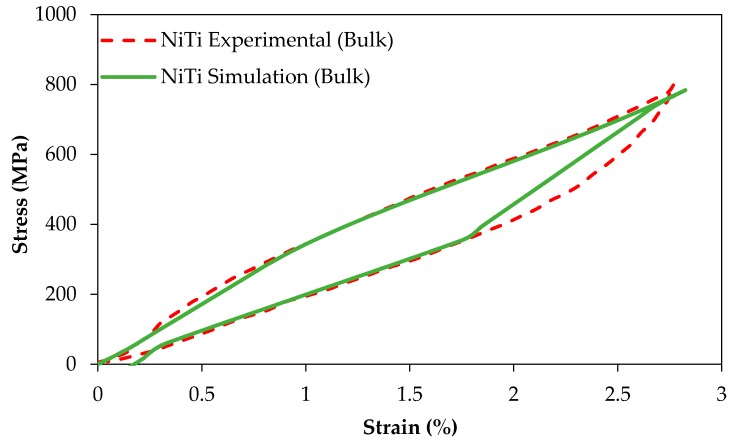
The simulation and experimental results of a compression test on dense NiTi.

Figure 9 compares the experimental and simulation results of the compression tests of dense (98%) cylindrical specimens (*r* > 0.99, *p* < 0.005, root-mean-square error (RMSE) = 12.6 MPa and *r* > 0.98, *p* < 0.005, RMSE = 38.3 MPa for the simulation of loading and unloading responses, respectively).

**Figure 10:**
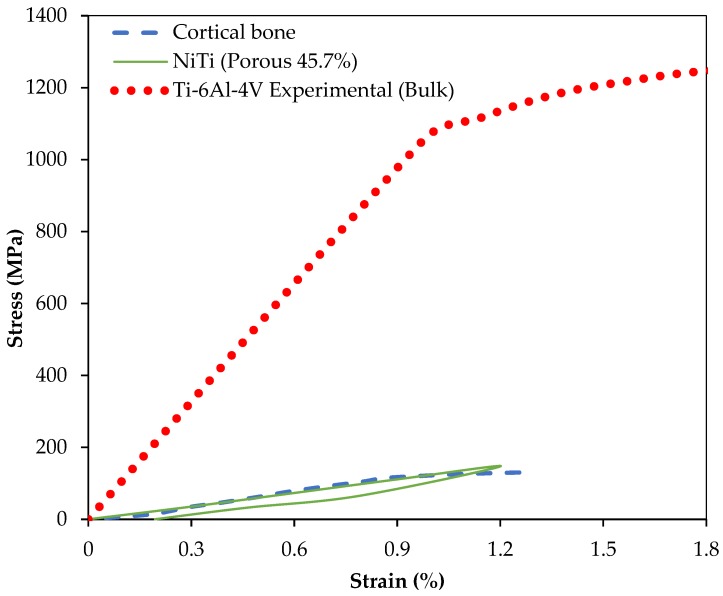
The equivalent stress–strain plot under compression for NiTi cubic samples with 45.7% porosity, cubes of dense Ti-6Al-4V [66] and samples of mandibular cortical bone [73].

The same UMAT was then used to simulate the behavior of porous superelastic parts. Figure 10 shows the compressive behavior of three different materials: cortical mandible bone (under compression) [73], NiTi cubes with 45.7% porosity, and Ti-6Al-V cubes [66]. We observed that the Ti-6Al-4V presents significantly higher stiffness than NiTi while the porous NiTi material shows similar stiffness to that of cortical bone (Figure 10). The results of this simulation suggest that a 45.7% porosity can be used to match the stiffness of NiTi parts to that of the cortical bone (i.e., 12 GPa) to which they are attached. This can also be done with porous Ti-6Al-4V with a very high level of porosity (see Section 6).

**Figure 11:**
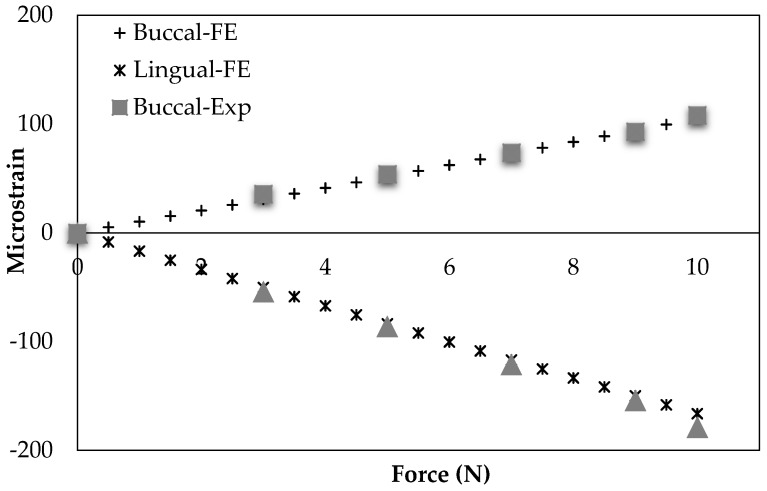
Model validation: a comparison between experimentally-obtained data (EXP) [74] with Finite Element Analysis (FEA)-predicted model data on the buccally and lingually placed strain gauges.

An experimental study by Ichim et al. group [74] is comparable to our healthy mandible model (model 1). They used a dry cadaveric mandible to measure the buccal and lingual strains in mandibular cortical bone using two strain gauges. The procedure and conditions of their experiment were applied to the FEA model used in this study. Figure 11 demonstrates their experimental outcomes as well as our simulation data. The correlation between our results and theirs were statistically significant (*r* > 0.99, *p* < 0.0005, RMSE = 2.8 × 10^−6^ (%) and *r* > 0.99, *p* < 0.0005, RMSE < 6.42 × 10^−6^ (%) for the Buccal and Lingual sides of mandible cortical bone in the molar region, respectively).

The results of our FEA models are presented at two different time points: (1) immediately following surgery, i.e., when bone fracture healing is occurring and the hardware is bearing most of the strain from chewing and other mandibular activities; and (2) post-healing where stiffness-matched fixation hardware is expected to increase the loading of the grafted bone over traditional, Ti-6Al-4V hardware, and drive the remodeling process [75,76]. One important consideration during healing period is to minimize the micromotion in the interfaces between the graft/host bone. This way, the risk of graft unvascularization caused by micromotion is minimized. Through a successful healing, the graft bone at the interfaces is integrated into the host mandible [4]. During the post healing period, the hardware should reduce the risk of stress shielding, which normally develops with the current standard of care titanium fixations.

**Figure 12:**
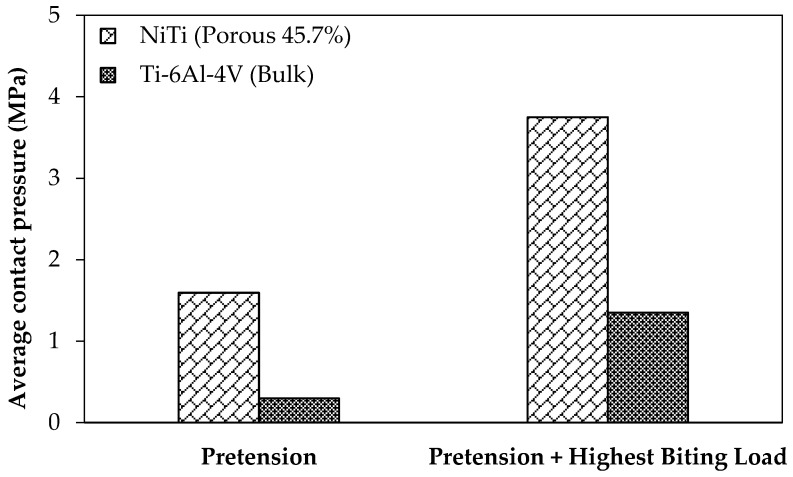
The effect of using NiTi and Ti-6Al-4V fixation plates on the average contact pressure at the interface between the graft and host bone (i.e., fibular bone graft and host mandible) during the healing period (i.e., in the immediate post-operative period there no strength at the graft-host bone junction).

Graft unvascularization is counted as one of the reasons for the mandibular reconstructive surgery failure. It is often caused by the relative movements, which are decreased as the contact pressure in the surfaces between the graft and the host bone increases [4]. For the loading regime during the healing and post-healing periods are studied with the current standard of care fixation hardware (Ti-6Al-4V, model 2) and the mandible reconstructed with stiffness-matched NiTi fixation hardware (model 3). It should be noted that for both cases pre-tension equal to 100 N is applied to the fixation hardware in order to increase the engagement. Two different loading scenarios are considered, at rest and under the highest occlusal load at M1. Figure 12 shows the associated average contact pressure in the interfaces between the graft and host bone.

In order to study the stress shielding effect, the von Mises stress distribution in the MRS was investigated using the current standard of care (model 2) and the mandible reconstructed with stiffness-matched NiTi (model 3). Both models were simulated in three different loading cases: case (A), applying highest biting force (i.e., 60% of the maximum bite force on the healthy mandible); case (B), applying a pure pretension (100 N) on each fixation; case (C), applying a pretension while the mandible is under highest biting force.

**Figure 13:**
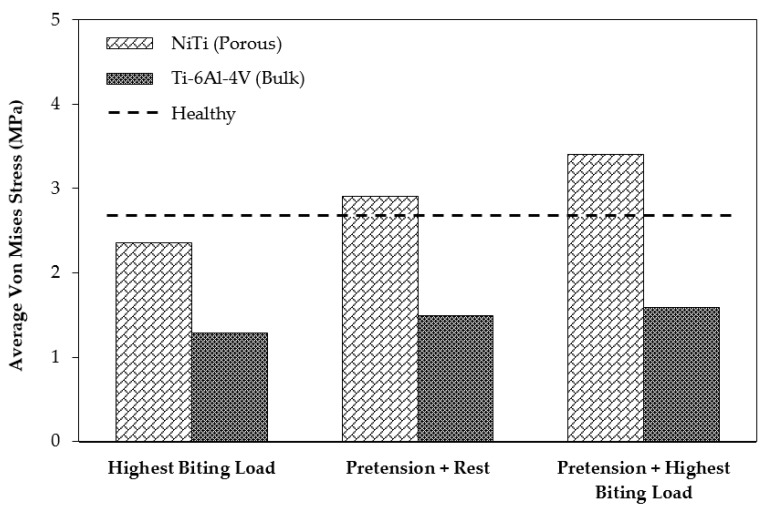
The average Von Mises stress on the surrounding bone in two cases of using Ti-6Al-4V and porous NiTi fixation plates, with and without applying the pretension to the fixation plates.

**Figure 14:**
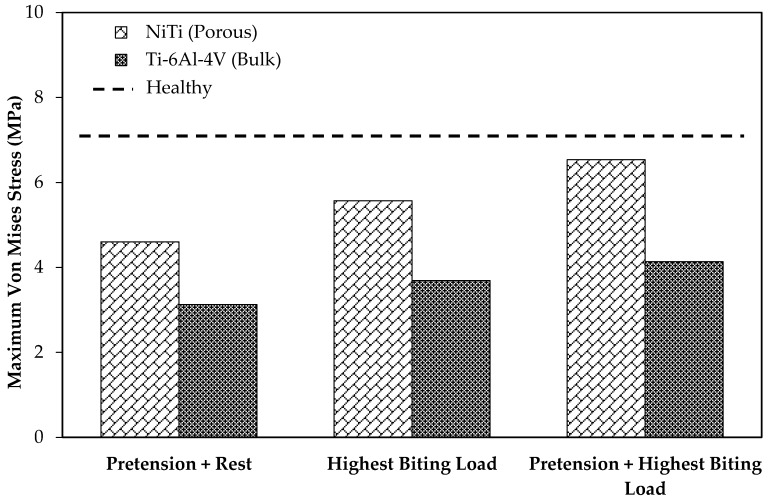
The maximum von Mises stress on the surrounding bone in two cases of using Ti-6Al-4V and porous NiTi fixation plates.

Figure 13 depicts the average von Mises stress for both fixations under the three loading scenarios. In a comparison with the traditional fixations, the results indicated higher average von Mises stresses on the cortical bone of the grafts in the case of using the porous NiTi fixations in all loading cases (by factors of 1.95, 1.82, and 2.14, respectively). In addition, these results were more adjusted to the average von Mises stress on the healthy mandible model (model 1), which indicates a more natural stress distribution on the grafted bone using the proposed fixations. Similarly, as shown in Figure 14, the maximum von Mises stress is higher when compared to Ti-6Al-4V fixation plates. It is worth noting that the stresses are all in a safe zone [73] (<100 MPa). It should be mentioned that the results of healthy mandible model (model 1) were attributed to the same region of the resected mandible (Figure 13 and Figure 14). The findings show that, by taking advantage of the superelasticity and applying pretension during the procedure, the von Mises stress on the fibular grafts bone increases, which results in a better long-term outcome for the patients. This increased stress can reduce the stress shielding effects and the risk of implant failure.

As Table 6 shows, the resultant maximum actual von Mises stress on the porous NiTi was higher than the dense traditional fixation plates in all loading cases. This stress for the NiTi implant at the worst cases (pretension and muscle forces) was 594 MPa, which is still in the safe region $\left(\sigma_{y}=1011\;\mathrm{MPa}\right)$ with the safety factor of 1.7.

As mentioned, a Young’s modulus of 12 GPa for NiTi fixation plates can be achieved by applying a specific percentage and type of porosity (i.e., 45.7% porosity). The unit cell of the structure is made out of three perpendicular cylinders with a diameter of 1.00 mm. As a step toward the manufacturing of patient-specific fixation plates, we used a previously healthy, dried cadaver mandible. To simulate the post healing period following reconstructive surgery. Two vertical fractures were created on the left M1-3 section of the mandible. Then a set of three porous NiTi fixation plates similar to the finite element model were designed based on the geometry derived from the CT-scan image of the dried mandible. Subsequently, they were fabricated using a Phenix PXM SLM machine.

**Figure 15:**
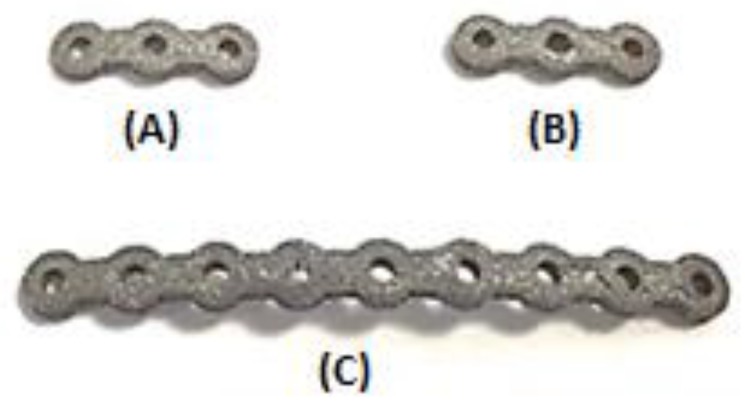
Additively manufactured: (**A**) superior mesial fixation plate; (**B**) superior distal fixation plate; and (**C**) inferior fixation plate (mandible bar).

**Figure 16:**
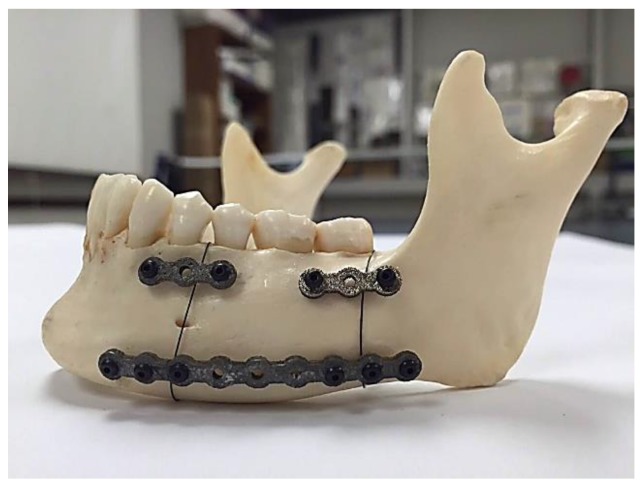
Reconstructed mandible using stiffness-matched, 3D printed, NiTi fixation devices.

Figure 15 shows the fabricated fixation plates. Since the fixation plates were designed based on the curvature of the host mandible, they were mounted on the mandible with no need to additional bending. Figure 16 shows these patient-specific fixation plates mounted on a dried cadaver mandible in order to immobilize the resected area.

## 6. Conclusions

Additive manufacturing methods have become popular for producing medical devices with high quality. The commonly used material for metallic implants, i.e., Ti-6Al-4V, presents high modulus of elasticity of 112 GPa, while the range of Young’s modulus of the bone varies in the range of 10–31.2 GPa. This high stiffness may result in implant failure due to the stress shielding effect, which results in un-vascularized bone. NiTi is a good candidate for biomedical devices because of lower stiffness (37 GPa), and the superelasticity behavior similar to that of the bone. The level of stiffness of NiTi can be tuned through introducing porosity to match the desired level.

**Figure 17:**
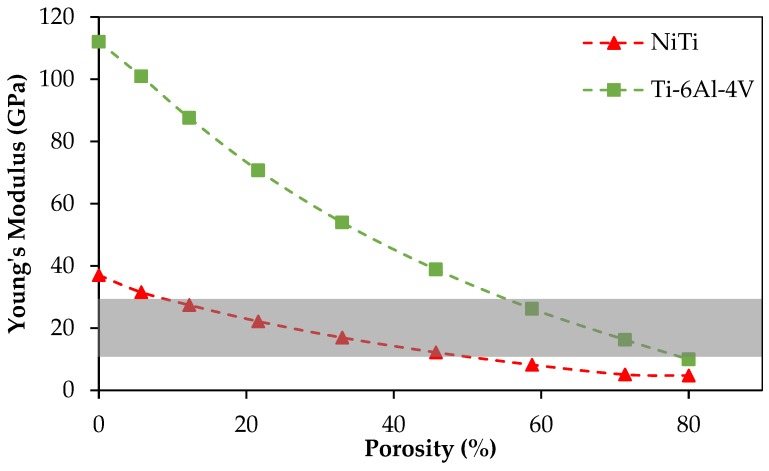
The effect of porosity on Young’s modulus for superelastic NiTi and Ti-6Al-4V. The range of bone stiffness is shown as highlighted zone.

**Figure 18:**
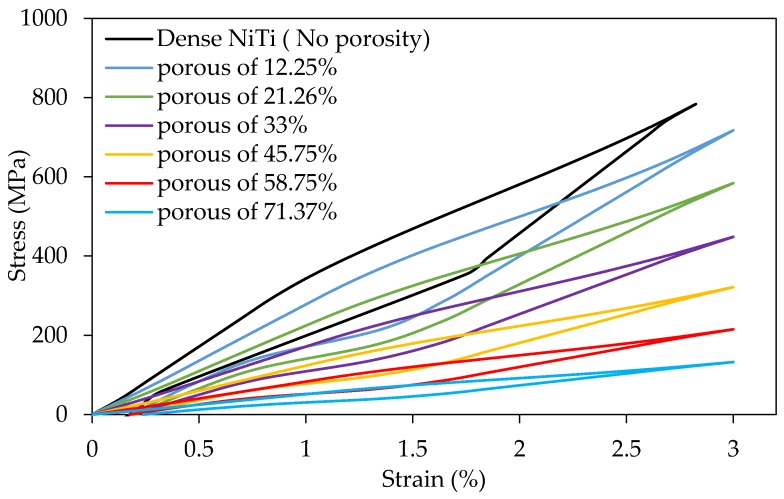
The effect of porosity on the compression behavior of superelastic NiTi; simulation results.

In our study, we focus on the design of a patient-specific stiffness-matched fixation hardware as a substitute for currently used fixations (Ti-6Al-4V). The purpose is to minimize the risk of implant failure (e.g., bone resorption, graft un-vascularization) and to produce the proposed fixation in a patient-specific manner. The fixation design is based on the exact shape and the required stiffness obtained by the CT-scan data of a dried mandible. Adding a 45.7% porosity to NiTi fixation hardware during 3D printing allows us to decrease the stiffness of fixation plates to stiffness of the surrounding bone (estimated from CT data). Figure 17 compares the Young’s modulus of NiTi with Ti-6Al-4V when similar porosity is introduced in both alloys. The NiTi alloys offer a better solution by reaching a low stiffness at a lower percentage porosity. In general, it is desirable to keep the level of porosity low to avoid the complications during the manufacturing and to avoid the possibility of failure due to stress concentration. Figure 18 shows the superelastic simulation results for NiTi parts with different levels of porosity.

In this work, we used finite element analysis to evaluate the effect of using this reduced stiffness for the post-surgical process. Based on this, two models of MRS were simulated in different loading scenarios. Our finite element results indicate a higher level of contact pressure (177%) during initial periods of healing due to pre-tension load in the case of using porous NiTi fixation plates. Such improvements can increase the chance of vascularization during the healing period [34,35]. Also, the results show that the average von Mises stress increased on host mandible (83%) and grafted bone (126%) for MRS simulation with porous NiTi implant. Considering stress distribution on the healthy mandible as a reference, the porous NiTi can cause better results compared to Ti-6Al-4V fixations.

These porous fixation plates may be fabricated using additive manufacturing (3D printing). For showing a patient-specific design for the fixation plates, a dried cadaver mandible was used. We successfully applied the design process for a case of reconstruction surgery on a dried cadaver mandible (patient-specific study). After the design stage, using Selective Laser Melting (SLM) we could fabricate these patient-specific fixation plates and assemble them on the dried mandible. These 3D printed, porous, NiTi fixation plates not only provide the required level of stiffness (12 GPa) and minimize the stress shielding probability, they also ensure hardware well-fitting that can be quickly placed, thereby reducing operation room time.

We have compared the use of NiTi and Ti-6Al-4V as the material for a patient-specific fixation plate. To this end, we created two different porous specimens with the same equivalent Young’s modulus of 12 GPa (estimated from CT scan data) using Ti-6Al-4V and Ni-rich NiTi. The required level porosity for the two specimens was different, 45.7% for the NiTi and 78.3% for the Ti-6Al-4V. Then we applied two percent equivalent strain (the recoverable strain by the cortical bone [14,15,16,73]) to both structures and reported the actual stress on the pore elements. The simulation results indicate that at in case of using porous Ti-6Al-4V the actual maximum stress (2981 MPa) exceeds the yield stress of the Ti-6Al-4V (970-1030 MPa) [66], and the porous structure failed (i.e., for the new and traditional porous fixation plates that have same equivalent stress-strain curve, a certain amount equivalent strain leads to much higher actual stress on the porous Ti-6Al-4V implants). Therefore, since it was not practical, we have not considered the use of a highly porous Ti-6Al-4V fixation. However, in the case of using NiTi for the porous specimen, thanks to the superelastic response of the material, this high value of equivalent strain (two percent) on the part can be completely recovered and the actual maximum stress (578 MPa) on the pore elements does not reach the yield stress $\left(\sigma_{y}=1011\;\mathrm{MPa}\right)$. Regarding this fact, the superelastic response of the Ni-rich NiTi makes it the proper choice for the patient-specific fixation plates.

## Figures and Tables

**Table 1:** The number of elements for the Finite Element Analysis model components. The number of tetrahedral elements was determined by convergence analysis.

Model Component	Number of Elements
Resected mandible	218,328
Teeth (13 total)	65,179
Ligaments (13 total)	21,053
Top graft	42,065
Lower graft	45,037
Fixation hardware(s)	58,327
Screws (10 total)	67,027

**Table 2:** Material properties of the Finite Element Analysis mandible components [1,47,49].

Material	$E_{x}$ (MPa)	$E_{y}$ (MPa)	$E_{z}$ (MPa)	${ʋ}_{xy}$	${ʋ}_{yz}$	${ʋ}_{xz}$
Cortical bone-symphysis region	23,000	15,000	10,000	0.3	0.3	0.3
Cortical bone-angle region	20,000	12,000	11,000	0.3	0.3	0.3
Cortical bone-rest of mandible	17,000	8200	6900	0.315	0.325	0.31
Cancellous bone	960	390	320	0.3	0.3	0.3
Cortical-fibular graft	26,800	26,800	26,800	0.3	0.3	0.3
Cancellous-fibular graft	1650	1650	1650	0.3	0.3	0.3
Teeth	17,600	17,600	17,600	0.25	0.25	0.25
Periodontal ligament	2.7	2.7	2.7	0.45	0.45	0.45
Ti-6Al-4V	112,000	112,000	112,000	0.3	0.3	0.3
NiTi	37,000–42,000 *	37,000–42,000 *	37,000–42,000 *	0.3	0.3	0.3

* More details are discussed in Section 4 and Section 5.

**Table 3:** Process parameters used in Selective Laser Melting (SLM) manufacturing of NiTi parts.

Effective Laser Power (W)	Layer Thickness (μm)	Scanning Velocity (m/s)	Hatch Distance (μm)	Energy Input (J/${\mathrm{mm}}^{3})$
250	30	1.25	120	55.5

**Table 4:** Mechanical properties of fabricated parts under compression test.

Material	E (GPa)	Yield Strength (MPa)
NiTi	37	1011 [3]
Ti-6Al-4V	112	970–1030 [66]

**Table 5:** Material parameters of dense Ni-rich NiTi fabricated by SLM technique at 37 °C. ($E_{A}$ and $E_{M}$ are the austenitic and martensitic modulus of elasticity). Transformation temperatures have been shown by $M_{s},M_{f},A_{s}$ and $A_{f}$.

Parameter	$E_{A}$ (GPa)	$E_{M}$ (GPa)	ν [72]	$M_{s}$ (K)	$M_{f}$ (K)	$A_{s}$ (K)	$A_{f}$ (K)
Value	37	42	0.33	263	243	270	280

**Table 6:** Maximum stress (MPa) on the fixation plates under different loading case.

Fixations Type	Highest Bite Loading	Pretension + REST	Pretension + Highest Biting Load
Porous NiTi fixation plate	328	485	594
Ti-6Al-4V fixation plate	98	132	299

## References

[1] Andani M.T., Moghaddam N.S., Haberland C., Dean D., Miller M.J., Elahinia M. (2014). Metals for bone implants. Part 1. Powder metallurgy and implant rendering. Acta Biomater..

[2] Elahinia M., Moghaddam N.S., Andani M.T., Amerinatanzi A., Bimber B.A., Hamilton R.F. (2016). Fabrication of NiTi through additive manufacturing: A review. Prog. Mater. Sci..

[3] Haberland C., Elahinia M., Walker J., Meier H. Visions, concepts and strategies for smart nitinol actuators and complex nitinol structures produced by additive manufacturing. Proceedings of the ASME 2013 Conference on Smart Materials, Adaptive Structures and Intelligent Systems.

[4] Amerinatanzi A., Zamanian H., Moghaddam N.S., Ibrahim H., Hefzy M.S., Elahinia M. On the Advantages of Superelastic Niti in Ankle Foot Orthoses. Proceedings of the ASME 2016 Conference on Smart Materials, Adaptive Structures and Intelligent Systems.

[5] Hadi A., Alipour K., Kazeminasab S., Amerinatanzi A., Elahinia M. Design and Prototyping of a Wearable Assistive Tool for Hand Rehabilitation using Shape Memory Alloys. Proceedings of the ASME 2016 Conference on Smart Materials, Adaptive Structures and Intelligent Systems.

[6] Mahtabi M., Shamsaei N., Mitchell M. (2015). Fatigue of nitinol: The state-of-the-art and ongoing challenges. J. Mech. Behav. Biomed. Mater..

[7] Mahtabi M., Shamsaei N., Rutherford B. (2015). Mean strain effects on the fatigue behavior of superelastic nitinol alloys: An experimental investigation. Procedia Eng..

[8] Mahtabi M., Shamsaei N. (2015). Multiaxial fatigue modeling for nitinol shape memory alloys under in-phase loading. J. Mech. Behav. Biomed. Mater..

[9] Esfahani S.N., Andani M.T., Moghaddam N.S., Mirzaeifar R., Elahinia M. (2016). Independent tuning of stiffness and toughness of additively manufactured titanium-polymer composites: Simulation, fabrication, and experimental studies. J. Mater. Process. Technol..

[10] Moghaddam N.S., Elahinia M., Miller M., Dean D. Enhancement of bone implants by substituting nitinol for titanium (Ti-6Al-4V): A modeling comparison. Proceedings of the ASME 2014 Conference on Smart Materials, Adaptive Structures and Intelligent Systems.

[11] Rahmanian R., Moghaddam N.S., Haberland C., Dean D., Miller M., Elahinia M. (2014). Load Bearing and Stiffness Tailored Niti Implants Produced by Additive Manufacturing: A Simulation Study. Behavior and Mechanics of Multifunctional Materials and Composites 2014.

[12] Hadi A., Qasemi M., Elahinia M., Moghaddam N. Modeling and experiment of a flexible module actuated by shape memory alloy wire. Proceedings of the ASME 2014 Conference on Smart Materials, Adaptive Structures and Intelligent Systems.

[13] Skoracki R., Miller M., Jahadakbar A., Taheri Andani M., Shayesteh Moghaddam N., Haberland C., Dean D., Walker J., Karaca H., Elahinia M. (2015). Additive Manufacturing of Nitinol Fixation Hardware for Reconstructing Mandibular Segmental Defects.

[14] Amerinatanzi A., Moghaddam N.S., Ibrahim H., Elahinia M. Evaluating a Niti Implant Under Realistic Loads: A Simulation Study. Proceedings of the ASME 2016 Conference on Smart Materials, Adaptive Structures and Intelligent Systems.

[15] Amerinatanzi A., Moghaddam N.S., Ibrahim H., Elahinia M. The Effect of Porosity Type on the Mechanical Performance of Porous Niti Bone Implants. Proceedings of the ASME 2016 Conference on Smart Materials, Adaptive Structures and Intelligent Systems.

[16] Moghaddam N.S., Amerinatanzi A., Saedi S., Turabi A.S., Karaca H., Elahinia M. Stiffness Tuning of Niti Implants Through Aging. Proceedings of the ASME 2016 Conference on Smart Materials, Adaptive Structures and Intelligent Systems.

[17] Habijan T., Haberland C., Meier H., Frenzel J., Wittsiepe J., Wuwer C., Greulich C., Schildhauer T., Köller M. (2013). The biocompatibility of dense and porous nickel–titanium produced by selective laser melting. Mater. Sci. Eng. C.

[18] Moghaddam N.S., Andani M.T., Amerinatanzi A., Haberland C., Huff S., Miller M., Elahinia M., Dean D. (2016). Metals for bone implants: Safety, design, and efficacy. Biomanuf. Rev..

[19] Es-Souni M., Es-Souni M., Fischer-Brandies H. (2005). Assessing the biocompatibility of NiTi shape memory alloys used for medical applications. Anal. Bioanal. Chem..

[20] Moghaddam N.S., Jahadakbar A., Elahinia M., Dean D., Miller M. (2015). The effect of adding dental implants to the reconstructed mandible comparing the effect of using Ti-6Al-4V and NiTi hardware. Tissue Engineering Part A.

[21] Martola M., Lindqvist C., Hänninen H., Al-Sukhun J. (2007). Fracture of titanium plates used for mandibular reconstruction following ablative tumor surgery. J. Biomed. Mater. Res. B Appl. Biomater..

[22] Spencer K., Sizeland A., Taylor G., Wiesenfeld D. (1999). The use of titanium mandibular reconstruction plates in patients with oral cancer. Int. J. Oral Maxillofac. Surg..

[23] Wei F.-C., Celik N., Yang W.-G., Chen I.-H., Chang Y.-M., Chen H.-C. (2003). Complications after reconstruction by plate and soft-tissue free flap in composite mandibular defects and secondary salvage reconstruction with osteocutaneous flap. Plast. Reconstr. Surg..

[24] Mariani P., Kowalski L., Magrin J. (2006). Reconstruction of large defects postmandibulectomy for oral cancer using plates and myocutaneous flaps: A long-term follow-up. Int. J. Oral Maxillofac. Surg..

[25] Moghaddam N., Ahmadi M., Webb J., Rahmani M., Sadegi H., Musavi M., Ismail R. (2012). Modeling of graphene nano-ribbon schottky diodes in the parabolic band structure limit. Proceedings of the Sixth Global Conference on Power Control and Optimization.

[26] Yilmaz M., Vayvada H., Menderes A., Demirdover C., Kizilkaya A. (2008). A comparison of vascularized fibular flap and iliac crest flap for mandibular reconstruction. J. Cranio-fac. Surg..

[27] Rahmani M., Ahmadi M., Kiani M.J., Ismail R. (2012). Monolayer graphene nanoribbon p–n junction. J. Nanoeng. Nanomanuf..

[28] Nagasao T., Miyamoto J., Tamaki T., Kawana H. (2010). A comparison of stresses in implantation for grafted and plate-and-screw mandible reconstruction. Oral Surg. Oral Med. Oral Pathol. Oral Radiol. Endodontol..

[29] Johansson B., Grepe A., Wannfors K., Hirsch J. (2001). A clinical study of changes in the volume of bone grafts in the atrophic maxilla. Dentomaxillofac. Radiol..

[30] Goh B.T., Lee S., Tideman H., Stoelinga P.J. (2008). Mandibular reconstruction in adults: A review. Int. J. Oral Maxillofac. Surg..

[31] Bagby G.W., Janes J.M. (1958). The effect of compression on the rate of fracture healing using a special plate. Am. J. Surg..

[32] Ayache N., Delingette H. (2003). Surgery simulation and soft tissue modeling. Proceeding of the International Symposium, IS4TM 2003.

[33] Elahinia M., Moghaddam N.S., Andani M.T., Skoracki R., Valerio I., Miller M., Dean D. (2015). Mitigating implant failure through design and manufacturing of nitinol fixation hardware. Tissue Engineering Part A.

[34] Binger T., Hell B. (1999). Resorption of microsurgically vascularized bone grafts after augmentation of the mandible. J. Cranio-Maxillofac. Surg..

[35] Li L., Blake F., Heiland M., Schmelzle R., Pohlenz P. (2007). Long-term evaluation after mandibular reconstruction with fibular grafts versus microsurgical fibular flaps. J. Oral Maxillofac. Surg..

[36] Bidabadi M., Natanzi A.H.A., Mostafavi S.A. (2012). Thermophoresis effect on volatile particle concentration in micro-organic dust flame. Powder Technol..

[37] Amerinatanzi A., Summers R., Ahmadi K., Goel V.K., Hewett T.E., Nyman E. (2016). A novel 3d approach for determination of frontal and coronal plane tibial slopes from mr imaging. The Knee.

[38] Kunchur M., Dean C., Moghadam N.S., Knight J., He Q., Liu H., Wang J., Lortz R., Sou I., Gurevich A. (2015). Current-induced depairing in the Bi_2_Te_3_/FeTe interfacial superconductor. Phys. Rev. B.

[39] Kunchur M.N., Dean C., Liang M., Moghaddam N.S., Guarino A., Nigro A., Grimaldi G., Leo A. (2013). Depairing current density of Nd_2−*x*_Ce*_x_*CuO_4−*δ*_ superconducting films. Phys. C Supercond..

[40] Rahmani M., Ahmadi M.T., Shayesteh N., Amin N.A., Rahmani K., Ismail R. Current-voltage modeling of bilayer graphene nanoribbon schottky diode. Proceedings of the 2011 IEEE Regional Symposium on Micro and Nanoelectronics (RSM).

[41] Schileo E., Dall’Ara E., Taddei F., Malandrino A., Schotkamp T., Baleani M., Viceconti M. (2008). An accurate estimation of bone density improves the accuracy of subject-specific finite element models. J. Biomech..

[42] Schileo E., Taddei F., Cristofolini L., Viceconti M. (2008). Subject-specific finite element models implementing a maximum principal strain criterion are able to estimate failure risk and fracture location on human femurs tested in vitro. J. Biomech..

[43] Zannoni C., Mantovani R., Viceconti M. (1999). Material properties assignment to finite element models of bone structures: A new method. Med. Eng. Phys..

[44] Taddei F., Pancanti A., Viceconti M. (2004). An improved method for the automatic mapping of computed tomography numbers onto finite element models. Med. Eng. Phys..

[45] Morgan E.F., Bayraktar H.H., Keaveny T.M. (2003). Trabecular bone modulus–density relationships depend on anatomic site. J. Biomech..

[46] Korioth T.W., Romilly D.P., Hannam A.G. (1992). Three-dimensional finite element stress analysis of the dentate human mandible. Am. J. Phys. Anthropol..

[47] Lovald S.T., Wagner J.D., Baack B. (2009). Biomechanical optimization of bone plates used in rigid fixation of mandibular fractures. J. Oral Maxillofac. Surg..

[48] Rahmani M., Ahmadi M., Webb J., Shayesteh N., Mousavi S., Sadeghi H., Ismail R. (2012). Trilayer graphene nanoribbon carrier statistics in degenerate and non degenerate limits. Proceedings of the Sixth Global Conference on Power Control and Optimization.

[49] Nagasao T., Miyamoto J., Kawana H. (2008). Biomechanical evaluation of implant placement in the reconstructed mandible. Int. J. Oral Maxillofac. Implant..

[50] Shetty P.P., Meshramkar R., Patil K.N., Nadiger R.K. (2013). A finite element analysis for a comparative evaluation of stress with two commonly used esthetic posts. Eur. J. Dent..

[51] Moghaddam N.S. (2015). Toward Patient Specific Long Lasting Metallic Implants for Mandibular Segmental Defects. Ph.D. Thesis.

[52] Moghaddam N.S., Jahadakbar A., Amerinatanzi A., Elahinia M., Miller M., Dean D. (2016). Metallic fixation of mandibular segmental defects: Graft immobilization and orofacial functional maintenance. Plast. Reconstr. Surg. Glob. Open.

[53] Tanaka E., Koolstra J. (2008). Biomechanics of the temporomandibular joint. J. Dent. Res..

[54] Amerinatanzi A., Moghaddam N.S., Jahadakbar A., Dean D., Elahinia M. On the effect of screw preload on the stress distribution of mandibles during segmental defect treatment using an additively manufactured hardware. Proceedings of the ASME 2016 11th International Manufacturing Science and Engineering Conference.

[55] Raad B., Moghaddam N.S., Elahinia M. (2016). A numerical simulation of the effect of using porous superelastic nitinol and stiff titanium fixation hardware on the bone remodeling. Nanosensors, Biosensors, and Info-Tech Sensors and Systems 2016.

[56] Saedi S., Turabi A., Taheri Andani M., Elahinia M., Karaca H. (2015). Thermo-mechanical characterization of NiTi alloys manufactured by selective laser melting. Proceedings of the ASME Conference on Smart Materials, Adaptive Structures and Intelligent Systems (SMASIS).

[57] Saedi S., Turabi A.S., Andani M.T., Haberland C., Karaca H., Elahinia M. (2016). The influence of heat treatment on the thermomechanical response of Ni-rich NiTi alloys manufactured by selective laser melting. J. Alloy. Compd..

[58] Ahmadi A., Mirzaeifar R., Moghaddam N.S., Turabi A.S., Karaca H.E., Elahinia M. (2016). Effect of manufacturing parameters on mechanical properties of 316l stainless steel parts fabricated by selective laser melting: A computational framework. Mater. Des..

[59] Ahmadi A., Moghaddam N.S., Elahinia M., Karaca H.E., Mirzaeifar R. Finite element modeling of selective laser melting 316l stainless steel parts for evaluating the mechanical properties. Proceedings of the ASME 2016 11th International Manufacturing Science and Engineering Conference.

[60] Shayesteh Moghaddam N., Dean D., Miller M., Elahinia M. Improving bone implant success by using nitinol as a substitute for titanium: A modeling comparison. Proceedings of the ASME 2014 Smart Materials, Adaptive Structures and Intelligent Systems.

[61] Elahinia M.H., Hashemi M., Tabesh M., Bhaduri S.B. (2012). Manufacturing and processing of NiTi implants: A review. Prog. Mater. Sci..

[62] Haberland C., Elahinia M., Walker J.M., Meier H., Frenzel J. (2014). On the development of high quality NiTi shape memory and pseudoelastic parts by additive manufacturing. Smart Mater. Struct..

[63] Dadbakhsh S., Speirs M., Kruth J.-P., Van Humbeeck J. (2015). Influence of SLM on shape memory and compression behaviour of NiTi scaffolds. CIRP Ann. Manuf. Technol..

[64] Dadbakhsh S., Speirs M., Kruth J.P., Schrooten J., Luyten J., Van Humbeeck J. (2014). Effect of SLM parameters on transformation temperatures of shape memory nickel titanium parts. Adv. Eng. Mater..

[65] Dadbakhsh S., Vrancken B., Kruth J.-P., Luyten J., van Humbeeck J. (2016). Texture and anisotropy in selective laser melting of NiTi alloy. Mater. Sci. Eng. A.

[66] Lee W.-S., Lin C.-F. (1998). Plastic deformation and fracture behaviour of Ti-6Al-4V alloy loaded with high strain rate under various temperatures. Mater. Sci. Eng. A.

[67] Mehrabi R., Kadkhodaei M., Ghaei A. (2012). Numerical implementation of a thermomechanical constitutive model for shape memory alloys using return mapping algorithm and microplane theory. Adv. Mater. Res..

[68] Mehrabi R., Kadkhodaei M., Andani M.T., Elahinia M. (2014). Microplane modeling of shape memory alloy tubes under tension, torsion, and proportional tension–torsion loading. J. Intell. Mater. Syst. Struct..

[69] Mehrabi R., Kadkhodaei M. (2013). 3D phenomenological constitutive modeling of shape memory alloys based on microplane theory. Smart Mater. Struct..

[70] Andani M.T., Haberland C., Walker J.M., Karamooz M., Turabi A.S., Saedi S., Rahmanian R., Karaca H., Dean D., Kadkhodaei M. (2016). Achieving biocompatible stiffness in NiTi through additive manufacturing. J. Intell. Mater. Syst. Struct..

[71] Andani M.T., Alipour A., Eshghinejad A., Elahinia M. (2013). Modifying the torque–angle behavior of rotary shape memory alloy actuators through axial loading: A semi-analytical study of combined tension–torsion behavior. J. Intell. Mater. Syst. Struct..

[72] Taheri A.M. (2015). Modeling, simulation, additive manufacturing, and experimental evaluation of solid and porous NiTi. Ph.D. Thesis.

[73] Van Eijden T. (2000). Biomechanics of the mandible. Crit. Rev. Oral Boil. Med..

[74] Ichim I., Kieser J., Swain M. (2007). Functional significance of strain distribution in the human mandible under masticatory load: Numerical predictions. Arch. Oral Biol..

[75] Raad B., Moghaddam N.S., Elahinia M. A comparison between porous NiTi and Ti-6Al-4V fixation hardware on bone remodeling after a reconstruction surgery. Proceedings of the ASME 2016 11th International Manufacturing Science and Engineering Conference.

[76] Elahinia M., Moghaddam N.S., Andani M.T., Rahmanian R., Walker J., Miller M.J., Dean D. (2014). Site-Specific Material Properties and the Additive Manufacturing of Nitinol Musculoskeletal Implants. Tissue Engineering Part A.

